# Calcineurin inhibitors differentially alter the circadian rhythm of T-cell functionality in transplant recipients

**DOI:** 10.1186/s12967-015-0420-5

**Published:** 2015-02-06

**Authors:** Sarah Leyking, Karin Budich, Kai van Bentum, Stephan Thijssen, Hashim Abdul-Khaliq, Danilo Fliser, Martina Sester, Urban Sester

**Affiliations:** Department of Internal Medicine IV, Saarland University, Homburg, Germany; Department of Transplant and Infection Immunology, Saarland University, D-66421 Homburg, Germany; Department of Paediatric Cardiology, Saarland University, Homburg, Germany; Current affiliation: Renal Research Institute, New York, USA

**Keywords:** Circadian rhythm, T-cell reactivity, Flow-cytometry, Whole blood assay, Kidney transplantation, Calcineurin inhibitor, Tacrolimus, Cyclosporine A, Immunosuppression, Pharmacokinetics, Pharmacodynamics

## Abstract

**Background:**

Graft survival in transplant recipients depends on pharmacokinetics and on individual susceptibility towards immunosuppressive drugs. Nevertheless, pharmacodynamic changes in T-cell functionality in response to drugs and in relation to pharmacokinetics are poorly characterized. We therefore investigated the immunosuppressive effect of calcineurin inhibitors and steroids on general T-cell functionality after polyclonal stimulation of whole blood samples.

**Methods:**

General T-cell functionality in the absence or presence of immunosuppressive drugs was determined in vitro directly from whole blood based on cytokine induction after stimulation with the polyclonal stimulus *Staphylococcus aureus* enterotoxin B. In addition, diurnal changes in leukocyte and lymphocyte subsets, and on T-cell function after intake of immunosuppressive drugs were analyzed in 19 patients during one day and compared to respective kinetics in six immunocompetent controls. Statistical analysis was performed using non-parametric and parametric tests.

**Results:**

Susceptibility towards calcineurin inhibitors showed interindividual differences. When combined with steroids, tacrolimus led to more pronounced increase in the inhibitory activity as compared to cyclosporine A. While circadian alterations in leukocyte subpopulations and T-cell function in controls were related to endogenous cortisol levels, T-cell functionality in transplant recipients decreased after intake of the morning medication, which was more pronounced in patients with higher drug-dosages. Interestingly, calcineurin inhibitors differentially affected circadian rhythm of T-cell function, as patients on cyclosporine A showed a biphasic decrease in T-cell reactivity after drug-intake in the morning and evening, whereas T-cell reactivity in patients on tacrolimus remained rather stable.

**Conclusions:**

The whole blood assay allows assessment of the inhibitory activity of immunosuppressive drugs in clinically relevant concentrations. Circadian alterations in T-cell function are determined by dose and type of immunosuppressive drugs and show distinct differences between cyclosporine A and tacrolimus. In future these findings may have practical implications to estimate the net immunosuppressive effect of a given drug-regimen that daily acts in an individual patient, and may contribute to individualize immunosuppression.

## Background

Outcome and survival of transplanted organs have significantly improved over the last decades due to the development of new immunosuppressive drugs. Optimal drug-dosing critically determines the balance between allograft rejection and infectious complications. Recommendations for drug-dosage are currently derived from empirically defined regimens evaluated in large cohort studies. In a given patient, the dosage is guided by determination of individual drug-levels in the blood, although this does not consider pharmacodynamic aspects of a particular drug or drug-combination or potential interindividual differences in drug-susceptibility [1]. A number of experimental approaches exist to monitor immunosuppressive therapy, although most approaches are not suitable for routine clinical use or have been performed on isolated blood cells under non-physiological conditions [2,3]. We have previously shown that analysis of T-cell functionality after stimulation of whole blood samples with viral or bacterial antigens allows for rapid quantitation of the potency of calcineurin inhibitors at clinically relevant concentrations [4,5]. The immunosuppressive effect was quantified by comparative analysis of intracellular cytokine induction in the presence or absence of immunosuppressive drugs [4,5]. The use of this stimulatory assay on T-cells specific for infectious antigens has the disadvantage that T-cell functionality may only be analyzed in individuals who have previously been in contact with the respective pathogen and who have mounted an appropriate immune response, and hence limits general use of this approach for every patient. In addition, suitability of this approach for characterizing interindividual differences in drug susceptibility and for analyzing inhibitory effects of drug combinations is largely unknown.

Apart from the direct inhibitory action of a given drug concentration, overall immune function in a patient after drug intake is determined by complex pharmacokinetics. We have recently shown that T-cell functionality in non-immunocompromised healthy individuals shows circadian variations that were inversely related to endogenous cortisol levels [6]. Peak levels of interferon (IFN)-γ and interleukin (IL)-2 from CD4 and CD8 T lymphocytes were found at night, when endogenous levels of cortisol were lowest [6]. Up to now, knowledge is limited as to how natural diurnal variation in immune function by endogenous cortisol is influenced by the presence of immunosuppressive drugs and their distinct pharmacokinetics in transplant recipients.

In this study, we therefore aimed at evaluating a flow-cytometric whole blood approach to characterize general T-cell functionality and to analyze susceptibility of an individual patient’s T-cell function towards immunosuppressive drugs. This approach was then used to characterize the inhibitory effect of calcineurin inhibitors and steroids on cellular immune function ex vivo, which was analysed for each individual drug alone as well as in combination. Moreover, the influence of pharmacokinetics assessed by drug plasma levels on circadian fluctuations in T-cell function were studied in renal transplant recipients with high and low immunosuppressive drug dosages and compared to the respective circadian rhythms in healthy controls.

## Methods

### Patients and controls

**Figure 1:**
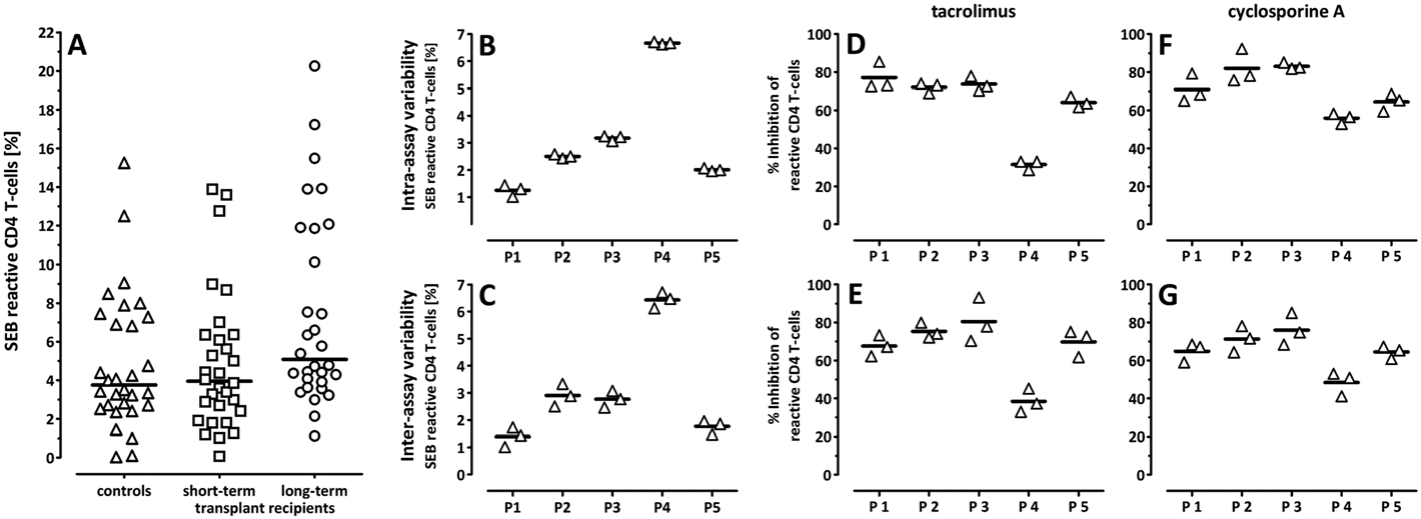
**Interindividual variability and intraindividual stability in T-cell function and different susceptibility towards calcineurin inhibitors. (A)** Inter-individual variability of SEB-reactive T-cell frequencies in 30 healthy controls (50.27 ± 13.5 years of age; 15 females), 30 short-term (51.71 ± 12.80 years of age; 15 females) and 30 long-term transplanted patients (50.16 ± 13.31 years of age; 14 females) is shown. Medians are indicated by horizontal lines and did not differ between the groups (Kruskal-Wallis test with Dunn’s post test). Intra- and inter-assay variability in SEB-reactive T-cell frequencies stimulated from blood samples of five healthy volunteers (P1-P5; 35.34 ± 5.54 years of age; 2 females) in the absence **(B and C)** or presence of 67 ng/ml tacrolimus **(D and E)** or 1111 ng/ml cyclosporine A **(F and G)**. Intra-assay variability of T-cell reactivity was determined by repeated measurements of the same blood sample **(B, D, and F)**. Inter-assay variability of T-cell reactivity was determined in three independently collected blood samples (0, two and six weeks; **C**, **E**, and **G**). The inhibitory effect on T-cells by calcineurin inhibitors was compared in relation to the samples treated without drugs. All drugs were solved in ethanol. Mean values are indicated as horizontal lines in panels **B**-**G**.

**Figure 2:**
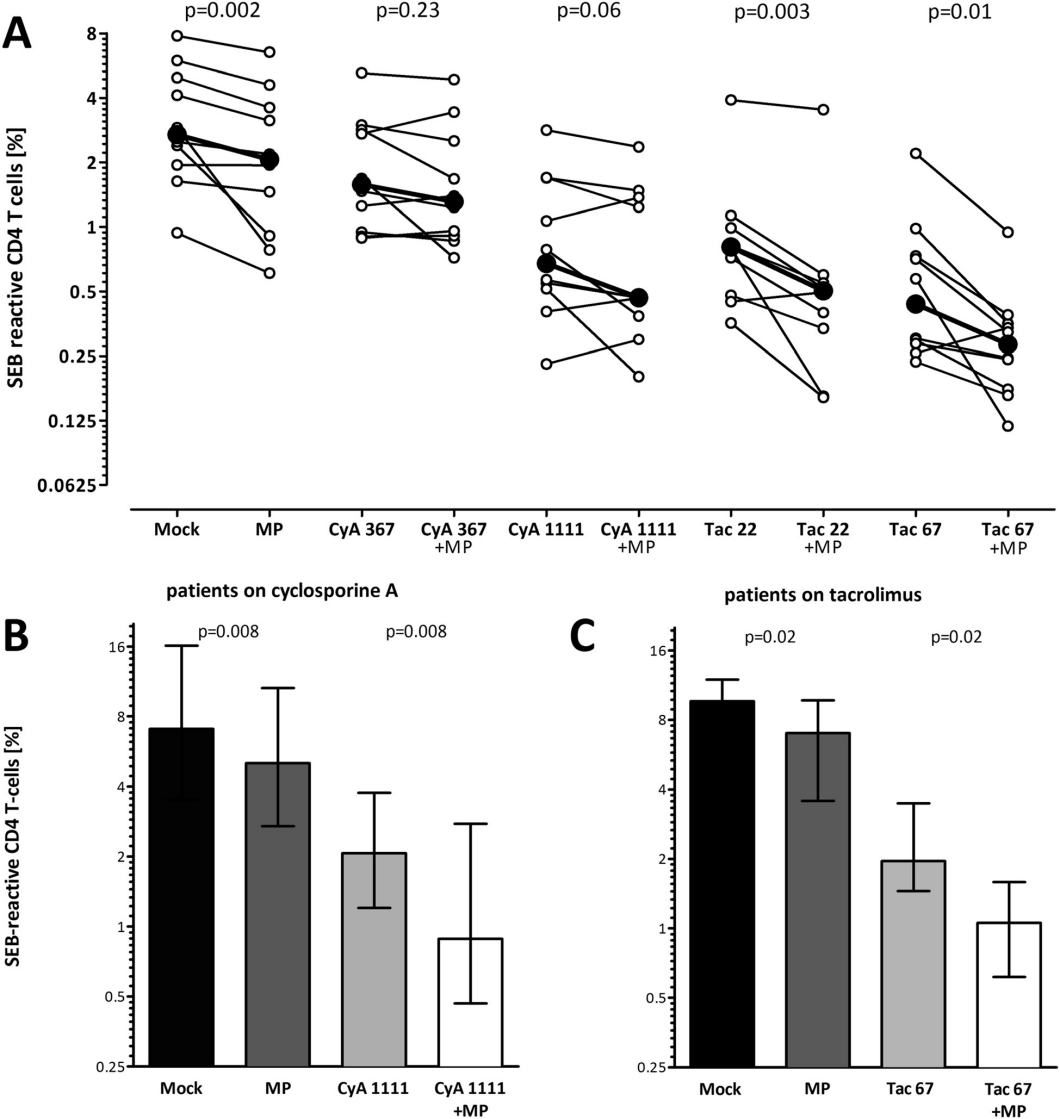
**Steroids show a combined inhibitory activity with tacrolimus and to a lesser extent with cyclosporine A. (A)** Whole blood samples of healthy individuals (n = 10; 22.3 ± 3.2 years; 6 females) were stimulated with SEB in the absence (Mock) or presence of cyclosporine A (CyA, 367 ng/ml and 1111 ng/ml, respectively) or tacrolimus (Tac, 22 ng/ml and 67 ng/ml, respectively) with and without steroids (MP). Bold dots represent the means connected by lines. **(B)** Whole blood samples of long-term transplanted patients on cyclosporine A (n = 8; 46.2 ± 12.8 years; 2 females) and **(C)** on tacrolimus (n = 8; 47.3 ± 13.4 years; 2 females) were stimulated with SEB in the absence of immunosuppressive drugs (mock) or high dose methylprednisolone (MP, 1000 ng/ml), cyclosporine A (1111 ng/ml) or tacrolimus (67 ng/ml) as well as respective combinations. Low dose calcineurin inhibitors were not analyzed in addition, as blood from patients already included trough levels due to immunosuppressive drug-treatment. Statistical analysis was performed using the Wilcoxon *t*-test. Data are expressed as median with interquartile range.

Characterization of *Staphylococcus aureus* enterotoxin B (SEB)-reactive T-cell frequency distributions was performed in 30 healthy controls, 30 short-term and 30 long-term renal transplant recipients matched for age and gender. Among them, subgroups were studied to analyze drug-susceptibility (details specified in the legends to Figures 1 and 2). For analyses of circadian variations, 6 healthy controls (49.3 ± 15.1 years; 2 females) and two groups of renal transplant recipients were recruited. The first group included 7 patients within the first month after transplantation (short-term; 59.1 ± 7.8 years; one female). The second group included 12 patients more than 5 months after transplantation (long-term; 51.4 ± 12.9 years; 7 females). Details on demographics and on immunosuppressive drugs of patients are specified in Table 1. Calcineurin inhibitors and mycophenolate mofetile (MMF) were taken twice daily in the morning and in the evening, whereas methylprednisolone and azathioprine were taken once daily in the morning. Long-term transplanted patients received steroid dosages between 2 and 10 mg and short-term transplanted patients between 12 and 36 mg. All patients had received anti-IL-2 receptor antibody induction. An 11 year-old female was recruited 9 years after heart transplantation; she received low dose prednisolone (2.5 mg twice daily) and the mTOR inhibitor everolimus (0.75 mg twice daily) and was converted from cyclosporine A (70 mg in the morning, 80 mg in the evening) to tacrolimus (2 mg in the morning and evening, respectively) due to recurrent rejection episodes and progressive impairment of kidney function. The steroid dose was the same before and after conversion.

**Table 1:** **Demographic characteristics of short-term and long-term transplanted patients analyzed for circadian variation of leukocyte numbers and T-cell function**

**Pt. #**	**Age [years]**	**Sex**	**Months post transplant**	**Category**	**Immunosuppressive medication**
1	49	Male	0.3	Short-term	Tac, MP, Aza
2	65	Male	0.4	Short-term	Tac, MP, Aza
3	63	Male	0.4	Short-term	Tac, MP
4	49	Male	0.4	Short-term	Tac, MP, Aza
5	57	Male	0.45	Short-term	Tac, MP, MMF
6	62	Female	0.5	Short-term	Tac, MP, Aza
7	69	Male	0.9	Short-term	CyA, MP
8	71	Female	5.6	Long-term	CyA, MP
9	39	Male	7.7	Long-term	Tac, MP
10	48	Female	11.3	Long-term	Tac, MP
11	56	Female	12.1	Long-term	Tac, MP, MMF
12	60	Female	15.6	Long-term	Tac, MP
13	49	Female	26.1	Long-term	CyA, Aza
14	62	Male	28.6	Long-term	MP, MMF
15	37	Male	28.7	Long-term	Tac, MP, MMF
16	59	Male	29.1	Long-term	MP, MMF
17	27	Female	57.3	Long-term	CyA, MP, MMF
18	41	Female	188.0	Long-term	CyA, MP
19	68	Male	253.8	Long-term	MP, MMF

CyA, cyclosporine A; Tac, tacrolimus; MMF, mycophenolate mofetile; MP, methylprednisolone; Aza, azathioprine; SD, standard deviation.

Heparinized blood samples for circadian analyses were collected at five time points spaced over a 24-hour period (8:00 a.m., 12:00 p.m., 8:00 p.m., 12:00 a.m. and 8:00 a.m.). Samples in the child were collected at 8:00 a.m., 10:00 a.m. and 12:00 p.m. Blood sampling at 8:00 a.m. was performed prior to the intake of immunosuppressive drugs. Subjects were woken up for the 12 a.m. blood collection but were otherwise on a regular sleep-wake rhythm during the period preceding the study. The study was approved by the local ethics committee (Ärztekammer des Saarlandes), and all individuals or the parents in the case of the child gave informed consent. The concentrations of tacrolimus and cyclosporine A were quantified by standard high-performance liquid chromatography.

### Quantitation of lymphocyte subpopulations and of T-cell functionality

The number of leukocytes and their subpopulations (neutrophils, monocytes, and lymphocytes) were determined from whole blood samples based on routine blood count and differential blood counts. Lymphocyte subpopulations were further quantified from whole blood after combined cell-surface staining using flow-cytometry. T-cell subpopulations were quantified by staining of CD3, CD4, and CD8 (all antibodies from BD, Heidelberg, Germany). B-cells and natural killer (NK)-cells were stained using CD19 (BD), CD3, and CD16 (Dako, Hamburg, Germany). T-cell functionality was determined after polyclonal stimulation of blood samples with 2.5 μg/ml SEB (Sigma, Deisenhofen, Germany), which elicits cytokine induction in both CD4 and CD8 T cells in all individuals [7-9]. For some experiments, blood samples were stimulated in the presence of immunosuppressive drugs. All stimulations were carried out at 37°C as described before [6] in the presence of 1 μg/ml co-stimulatory antibodies against CD28 and CD49d (BD). After 2 hours, cytokines were accumulated intracellularly using 10 μg/ml brefeldin A. After a total of 6 hours, leukocytes were fixed and erythrocytes were lysed using BD-lysing solution. Thereafter, cells were treated with 0.1% saponin (Sigma) for permeabilization of leukocytes and removal of remaining erythrocytes, and immunostained in FACS buffer (PBS, 5% FCS, 0.5% bovine serum albumin, 0.07% NaN_3_) in the presence of 0.1% saponin for 30 min at room temperature using saturating concentrations of anti-CD4 and anti-CD69 in combination with anti-IFN-γ or anti-IL-2 (all antibodies from BD). CD8 T-cell reactivity was estimated based on cells reacting in the CD4 T-cell negative lymphocyte fraction. Samples were analyzed on a FACScan or FACS-Canto II using Cellquest Pro or DIVA-software (BD).

### Statistical analysis

Statistical analysis was performed using Prism-v5.03 software (Graphpad). Differences in SEB-reactive CD4 T-cell frequencies were analyzed using the Kruskal-Wallis test with Dunn’s post-test. The effect of immunosuppressive drugs on T-cell functionality was analyzed using the Wilcoxon test. Differences in cell numbers, T-cell reactivity, and drug-levels during the 24 hour period from normalized data were analyzed using repeated measures ANOVA with Tukey’s post-test.

## Results

### Inhibition of cytokine induction to quantify the potency of calcineurin inhibitors

To establish an assay to study the effect of immunosuppressive drugs on T-cell functionality on an individual basis, whole blood samples were stimulated with SEB, which leads to polyclonal stimulation of both CD4 and CD8 T-cells [7-9]. T-cell functionality was subsequently assessed by quantitation of activated CD69 positive IFN-γ producing cells. This polyclonal stimulus was chosen as it elicited a cytokine response in all individuals tested. As exemplified for CD4 T-cells, SEB-reactive T-cells were detectable in both controls and patients, and median percentages did not differ between the groups when blood samples were analyzed at trough levels prior to intake of immunosuppressive drugs (Figure 1A). However, as reactive T-cell frequencies showed a considerable interindividual variability ranging from 0.025-20.25%, we first used blood samples of five healthy controls (P1-P5) to study intra-assay and inter-assay variability in the individual percentage of SEB-reactive CD4 T-cells. As shown in Figure 1B, three independent stimulatory reactions performed from the same samples of a given individual yielded similar frequencies of reactive CD4 T-cells (intra-assay variability). This also held true for results from three independent blood samples drawn at different time points (0, two and six weeks, inter-assay variability, Figure 1C). Thus, although interindividual variability of SEB-reactive T-cell frequencies was high (Figure 1A), the percentage of SEB-reactive T-cells in a given individual was stable. This stability was considered as an essential prerequisite to subsequently use this stimulatory approach to quantify the inhibitory effects of immunosuppressive drugs.

To assess intra-individual variability in the susceptibility towards immunosuppressive drugs, the same blood samples were treated with either 67 ng/ml tacrolimus or 1111 ng/ml cyclosporine A. As with overall frequencies of SEB-reactive CD4 T-cells, the inhibitory effect of the two drugs on T-cell function only showed low intra- (<5.0%) or inter-assay coefficients of variation (<6.2%, Figure 1D-G). Of note, however, the inhibitory effect of a drug did show interindividual variability. The difference in drug-susceptibility was similar for both calcineurin inhibitors and was most striking for control subject P4, who had the highest percentage of reactive CD4 T-cells and whose reactivity was least affected by either drug. Similar results were found for CD4 negative lymphocytes that were quantified as an estimate of CD8 T-cell reactivity (intra- <5.0% and inter-assay coefficient of variation <8.1%, data not shown). Taken together, susceptibility towards calcineurin inhibitors shows inter-individual differences. The low intra- and inter-assay variability suggests that the cytokine-based whole blood assay may be used to reliably quantify the effect of immunosuppressive drugs on an individual basis.

### The two calcineurin inhibitors show differential inhibitory effects in combination with steroids

To analyze the influence of immunosuppressive drug-combinations on T-cell function after SEB-stimulation, blood samples from ten healthy controls were treated with or without 1 μg/ml methylprednisolone in the presence or absence of increasing doses of tacrolimus (22 ng/ml and 67 ng/ml) or cyclosporine A (367 ng/ml and 1111 ng/ml; Figure 2A). Drug concentrations were titered to achieve an equal inhibition of SEB-reactive T-cells (data not shown). Steroid treatment alone only led to a median reduction in SEB-reactive CD4 T-cells by 23.5% (interquartile range (IQR) 30.1%, p = 0.002), whereas inhibition by the two calcineurin inhibitors was more pronounced (by 65.6%, IQR 23.1%, for 1111 ng/ml cyclosporine A, and by 82.0%, IQR 15.7%, for 67 ng/ml tacrolimus compared to mock treatment). Interestingly, when either 367 or 1111 ng/ml cyclosporine A were combined with steroids, there was a slight additional inhibition, but this was not significantly different from cyclosporine A treatment alone (p = 0.23 and p = 0.06, respectively). Of note, the inhibitory effect was more pronounced for combined treatment with tacrolimus and steroids, as the combination of either 22 or 67 ng/ml tacrolimus with steroids led to a significant further decrease in reactive CD4 T-cells compared to tacrolimus alone (p = 0.003 and p = 0.01, respectively, Figure 2A). Thus, all three drugs reduced SEB-reactive CD4 T-cell frequencies. The net inhibitory effect of steroids and tacrolimus was more pronounced as compared to that between steroids and cyclosporine A.

The combined inhibitory action of immunosuppressive drugs was also analyzed in samples from long-term transplant recipients on a cyclosporine A or tacrolimus based drug-regimen (8 patients in each group). To keep the basal effect of regular drug-intake to a minimum, whole blood samples were drawn at trough-levels in the morning and stimulated with SEB in the presence or absence of additional 1 μg/ml methylprednisolone with or without high-dose cyclosporine A (1111 ng/ml, Figure 2B) or tacrolimus (67 ng/ml, Figure 2C). Methylprednisolone treatment alone led to a similar median decrease in T-cell reactivity in both groups (by 23.0%, IQR 27.2%, in patients on cyclosporine A, and by 25.8%, IQR 16.1%, in patients on tacrolimus). In line with results from controls (Figure 2A), the decrease induced by the two calcineurin inhibitors was more pronounced (by 71.9%, IQR 24.8% and by 76.8%, IQR 18.5%, respectively, Figure 2B and C). Likewise, the combined treatment of samples with steroids and cyclosporine A or tacrolimus caused a stronger inhibition (by 85.9% (IQR 10.4%) and 87.1% (IQR 15.0%), respectively) compared to treatment with the respective calcineurin inhibitor alone (p = 0.008 and p = 0.02, respectively). Thus, all three drugs led to a significant reduction in SEB-reactive CD4 T-cell frequencies in both controls and patients and combination of calcineurin inhibitors with steroids increased the inhibitory effect over that observed for each drug alone.

### Controls and transplant recipients differ in the circadian rhythm of cell counts

**Figure 3:**
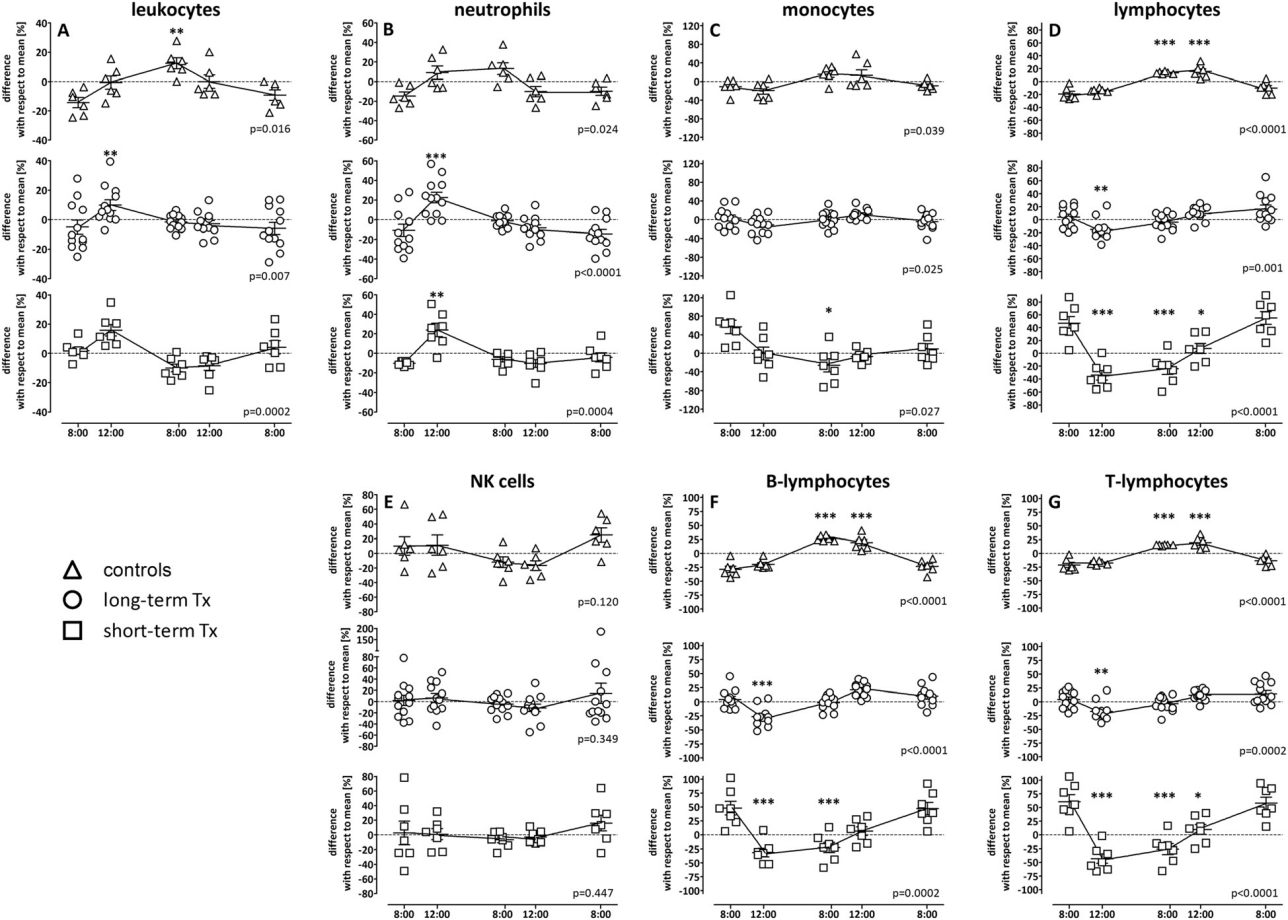
**Controls and transplant recipients differ in the circadian rhythm of cell counts.** Circadian rhythm of **(A)** leukocytes and their subpopulations, namely **(B)** neutrophils, **(C)** monocytes, **(D)** lymphocytes and their subpopulations **(E)** NK-cells, **(F)** B- and **(G)** T-lymphocytes in peripheral blood of healthy controls (n = 6), long-term (n = 12) and short-term transplanted patients (n = 7) was determined over 24 hours at 8:00 a.m., 12:00 p.m., 8:00 p.m., 12:00 a.m. and the following day at 8:00 a.m. Shown are the differences in cell numbers with respect to the mean that was calculated from all values analyzed over the 24 h-time period (stippled line). The variance of cell populations at each time point with respect to this 24 h-mean is expressed as mean ± standard error of the mean. Statistical analysis was performed using the repeated measures ANOVA with Tukey’s post-test. Statistically significant differences are indicated with respect to the initial 8:00 a.m. sample. * p < 0.05, ** p < 0.01, *** p < 0.001.

As both T-cell numbers and their functionality are influenced by circadian rhythm [6], we first studied the influence of iatrogenic immunosuppression on circadian variation of leukocyte numbers and their subsets. To study the effect of different overall drug-levels, the respective cell numbers were quantified in short-term and long-term transplant recipients and compared to controls. To allow for a robust analysis of cell populations over time, cell numbers were normalized with respect to the daily mean that was calculated from all values from one individual over the 24 h time period as described before [6]. As shown in Figure 3, the circadian rhythm of cell counts showed a pronounced variation over time and differed between the three groups. Leukocytes of controls had their nadir at 8:00 a.m. and their highest peak at 8:00 p.m. (p = 0.016), whereas the leukocyte peak in transplant patients was already observed at 12:00 noon, and the increase was only significant in long-term-transplanted patients (p = 0.007). Long-term transplanted patients had their nadir at 8:00 in the morning, whereas lowest leukocyte counts in short-term transplant patients were found at 8:00 in the evening (Figure 3A). When analyzing leukocyte subpopulations, a striking circadian variation was seen in neutrophils and lymphocytes, albeit with completely different kinetics. The variation in neutrophil numbers resembled that of overall leukocytes in all three groups (Figure 3B). In contrast, lymphocyte numbers in transplant recipients showed a remarkable drop four hours after intake of immunosuppressive drugs (at 12:00 p.m., Figure 3D). This drop was more pronounced in short-term transplant recipients and levels remained considerably low until 8:00 p.m. Among lymphocyte subpopulations, NK-cell numbers only showed marginal circadian variations (Figure 3E), whereas patterns of B- and T-cells largely resembled that of overall lymphocytes (Figure 3F and G). Taken together, circadian variations in leukocyte numbers and their subpopulations differed between controls and transplant recipients. In line with increased levels of immunosuppressive drugs, variations over time were more pronounced in short-term transplant recipients.

### Differential circadian rhythm of T-cell function in transplant recipients and healthy controls

**Figure 4:**
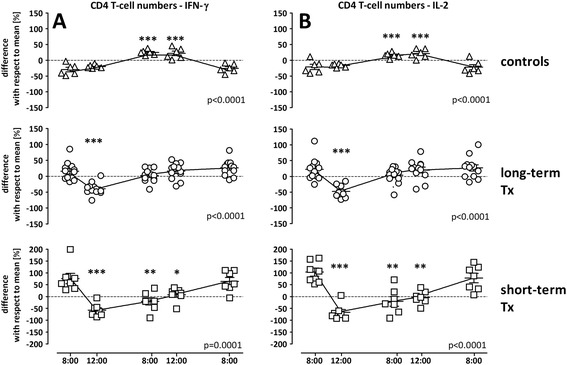
**Differential circadian rhythm of T-cell function in transplant recipients and healthy controls.** Circadian rhythm of SEB-reactive CD4 T-cell numbers in peripheral blood of healthy controls (n = 6), long-term (n = 12) and short-term transplanted patients (n = 7) was determined over 24 hours at 8:00 a.m., 12:00 p.m., 8:00 p.m., 12:00 a.m. and the following day 8:00 a.m. Shown are the differences in absolute cell numbers of IFN-γ positive **(A)** and IL-2 positive **(B)** CD4 T-cells with respect to the mean that was calculated from all values analyzed over the 24 h-time period (stippled line). The variance of cytokine-producing T-cells at each time point with respect to this 24 h-mean is expressed as mean ± standard error of the mean. Statistical analysis was performed using the repeated measures ANOVA with Tukey’s post-test. Statistically significant differences are indicated with respect to the initial 8:00 a.m. sample. *p < 0.05, **p < 0.01, ***p < 0.001.

To assess circadian changes in T-cell functionality in controls and transplant recipients over a 24 h time period, blood samples were stimulated with SEB and analyzed for the induction of CD69 and the cytokines IFN-γ and IL-2. In controls, the number of IFN-γ producing T-cells showed a continuous increase towards the evening (by 55.9%, IQR 13.2%) and midnight (by 51.1%, IQR 31.4%, p < 0.0001, Figure 4A). This effect was also observed for cells expressing IL-2, although the median increase was less pronounced (by 44.0%, IQR 27.6%, p < 0.0001, Figure 4B). In contrast, median numbers of IFN-γ producing CD4 T-cells in transplant recipients showed a significant decrease already at 12 p.m. (4 hours after intake of the morning medication) in both long-term (by 57.6%, IQR 16.5%, p < 0.0001) and short-term transplant recipients (by 120.8%, IQR 32.0%, p = 0.0001; Figure 4A). Higher drug-levels in short-term transplanted patients were associated with a prolonged decrease in the absolute numbers of SEB-reactive CD4 T-cells and this effect was also observed for CD4 T-cells producing IL-2. Again, similar kinetics were found for SEB-reactive cells among CD4-negative lymphocytes, which may serve as estimate of CD8 T-cell reactivity, and dynamics in percentage of cytokine-producing T cells resembled those of absolute T cell numbers (data not shown). Together with kinetics shown in Figure 3G, these data indicate that both the numbers of T-cells and their functionality differ in patients and controls, and dynamics in T-cell functionality were more pronounced in short-term transplant recipients.

### Cyclosporine A and tacrolimus differentially affect circadian rhythms in T-cell reactivity

**Figure 5:**
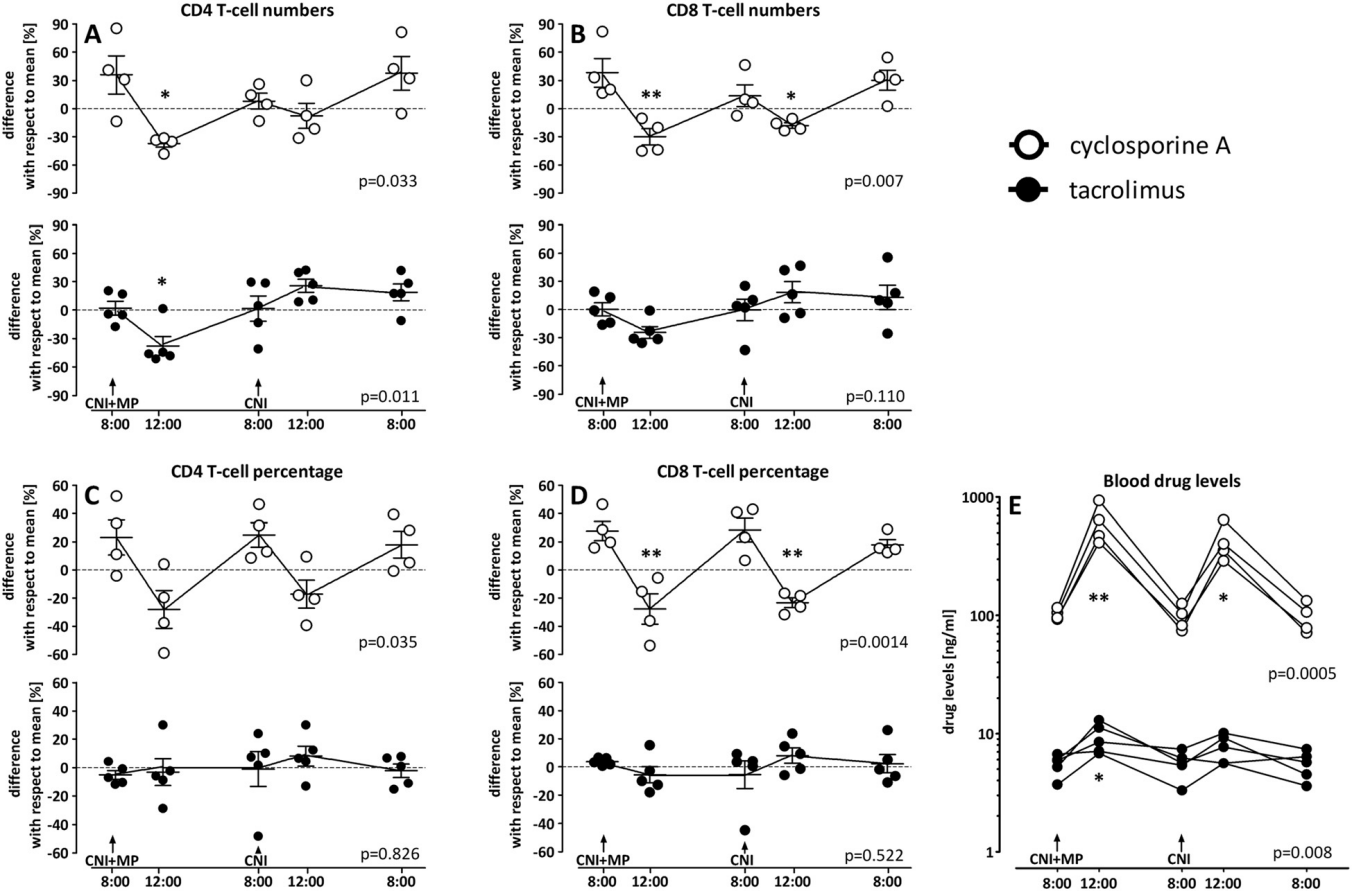
**Cyclosporine A and tacrolimus differentially affect circadian rhythms in T-cell reactivity.** Circadian rhythm of SEB-stimulated CD4 and CD8 T-cells in the peripheral blood of long-term transplanted patient taking cyclosporine A (n = 4) or tacrolimus (n = 5) determined over 24 hours at 8:00 a.m., 12:00 p.m., 8:00 p.m., 12:00 a.m. and the following day 8:00 a.m. Shown are the differences in absolute cell numbers of IFN-γ positive **(A)** CD4 and **(B)** CD8 T-cells with respect to the mean that was calculated from all values analyzed over the 24 h-time period (stippled line). In addition, relative CD4 **(C)** and CD8 **(D)** T-cell frequencies are shown. The variance of cytokine-producing T-cells at each time point with respect to this 24 h-mean is expressed as mean ± standard error of the mean. **(E)** Corresponding levels of calcineurin inhibitors in ng/ml determined from peripheral blood of the patients. Statistical analysis was performed using the repeated measures ANOVA with Tukey’s post-test. Statistically significant differences are indicated with respect to the 8:00 a.m. and 8:00 p.m. samples after drug-intake. The intake of calcineurin inhibitors (CNI) alone or in combination with methylprednisolone (MP) is indicated. *p < 0.05, **p < 0.01.

To analyze potential differences in the inhibitory action of the two calcineurin inhibitors during the 24 h period, circadian variations in T-cell functionality were separately analyzed in long-term transplant recipients who received two daily doses of either cyclosporine A or tacrolimus in combination with methylprednisolone once daily. Interestingly, the absolute numbers of IFN-γ reactive T-cells in patients on cyclosporine A showed biphasic kinetics where levels were lowest 4 hours after drug-intake (p = 0.033, Figure 5A). A similar effect was observed for CD8 T-cell numbers (p = 0.007, Figure 5B), and when the inhibitory effect was quantified as dynamic changes in relative T-cells producing IFN-γ (Figure 5C and D). In marked contrast, CD4 and CD8 T-cell numbers in patients on tacrolimus exhibited only a slight decrease at 12 p.m. (Figure 5A and B). Despite a second dose of tacrolimus at 8 p.m., the numbers of reactive T-cells even increased thereafter and stabilized at 12:00 a.m. and the early morning hours. Of note, unlike marked biphasic kinetics in patients on cyclosporine A, the percentages of SEB-reactive CD4 and CD8 T-cells in patients on tacrolimus were considerably stable during the 24 h-interval (p = 0.826 and p = 0.522, respectively, Figure 5C and D). Similar kinetics were found for CD4 T-cells producing IL-2 (data not shown). Interestingly, in line with inhibitory effects on T-cell reactivity, corresponding cyclosporine A levels 4 h after drug-intake in the morning and in the evening showed a strong increase (6.01 and 4.37-fold, respectively). In contrast, respective levels of tacrolimus only increased 1.89-fold in the morning and 1.28-fold in the evening (Figure 5E).

**Figure 6:**
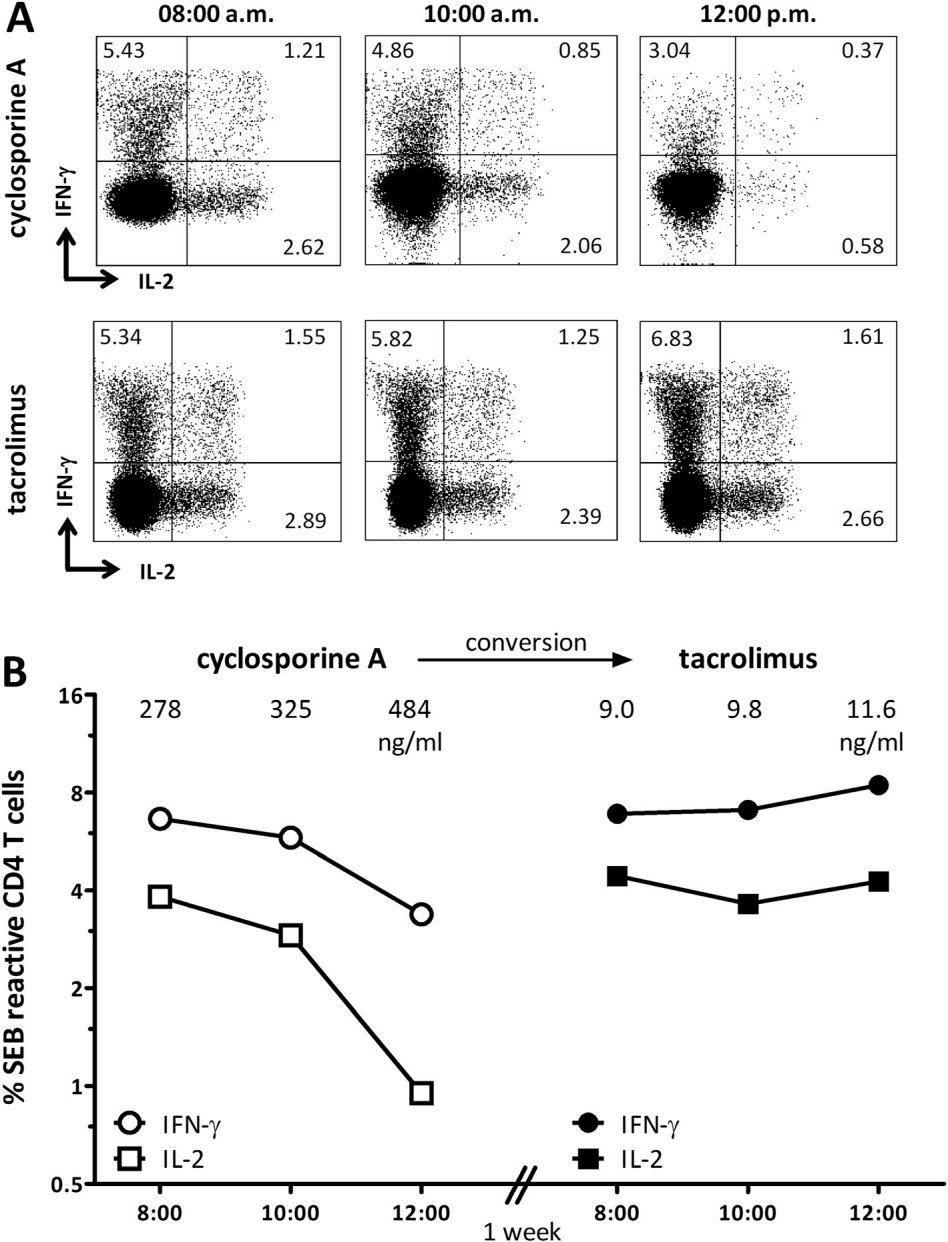
**T-cell function after drug-intake shows different dynamics after conversion from cyclosporine A to tacrolimus. (A)** Dot plots above show SEB-stimulated CD4 T-cells producing IL-2 (upper left quadrant) or IFN-γ (lower right quadrant) or both cytokines (upper right quadrant) before (8:00 a.m.) and after (10:00 a.m. and 12:00 p.m.) intake of cyclosporine A (upper panel) or tacrolimus (lower panel). Percentages of reactive CD4 T-cells are indicated in the corresponding quadrants. Kinetics of SEB-reactive T-cells producing the cytokine IFN-γ (circles, corresponding to cells in the two upper quadrants) and IL-2 (squares, corresponding to cells in the two right quadrants) at 8:00 a.m., 10:00 a.m. and 12:00 p.m. under cyclosporine A and after conversion to tacrolimus are shown in panel **(B)**. Corresponding drug-levels are indicated.

To exclude that the difference in the inhibitory effects of the two calcineurin inhibitors were due to variations in drug-susceptibility of an individual patient, we took the chance to study a case of an 11 year-old heart transplanted girl before and after conversion from cyclosporine A to tacrolimus to due to recurrent rejection episodes and progressive decline of kidney function. Conversion was clinically indicated and offered a unique opportunity to analyze the effect of cyclosporine A and tacrolimus on T-cell cytokine induction in two series of three sequential whole blood samples drawn from one patient. In line with other patients on cyclosporine A (Figure 5), the percentage of SEB-reactive CD4 T-cells producing IFN-γ, IL-2 or both cytokines decreased 4 hours after intake of cyclosporine A, whereas respective T-cell frequencies remained stable after intake of tacrolimus (Figure 6A and B). Corresponding levels of cyclosporine A showed a 1.74-fold increase (from 278 to 484 ng/ml), whereas the respective tacrolimus levels showed a 1.28-fold increase only (from 9 to 11.6 ng/ml). Thus, although only based on one case, these data further corroborate the differential effect of the two calcineurin inhibitors on the circadian rhythm of T-cell functionality.

## Discussion

Overall immune function in patients after transplantation may be influenced by both immunosuppressive drugs as well as by the endogenous circadian rhythm of the immune response. In this study, we show that T-cell function and individual susceptibility towards immunosuppressive drugs can be analyzed directly from whole blood. When analyzing drug-combinations ex vivo, a combined inhibitory activity was observed for steroids in combination with tacrolimus but to a lesser extent in combination with cyclosporine A. Moreover, transplant recipients on a calcineurin inhibitor-based drug-regimen show marked circadian variations in both T-cell numbers and function that differ from those found in healthy controls. Whereas the circadian rhythm of T-cell numbers and function in controls mainly depend on endogenous cortisol production [10], the circadian rhythm in transplant recipients is distinctly altered by the type and level of immunosuppressive drugs. Interestingly, as with in vitro analyses, the two calcineurin inhibitors differ in their circadian effect on T-cell function when combined with steroids in vivo. Whereas T-cell function in patients on a cyclosporine A-based regimen showed pronounced biphasic kinetics with nadirs 4 h after intake of the respective morning and evening doses, T-cell functionality in patients on tacrolimus was rather stable over a period of 24 hours and only showed a slight suppression after drug-intake in the morning.

This study explored the use of a pharmacodynamic approach of immunosuppressive drug-monitoring, where whole blood cells were induced to produce cytokines after polyclonal stimulation in the presence or absence of individual drugs or their combinations. Although viral antigens are also suitable stimuli to assess the inhibitory action of immunosuppressive drugs [6,5,4], SEB was chosen as a universally applicable stimulus that elicited a T-cell response in all patients. In addition, T-cell frequencies stimulated by SEB were generally higher than those observed after pathogen-specific immunity which allows for a better characterization of the inhibitory effects by immunosuppressive drugs. Of note, as with other stimuli, the net percentage of SEB-reactive T-cells per se does not directly provide any information on individual immune function, as SEB-reactive T-cell frequencies are interindividually variable. Instead, changes in SEB-reactive T-cell functionality in response to immunosuppressive drugs for each individual should be related to T-cell frequencies determined at trough levels or in the absence of drugs or dynamic changes should be assessed over time in relation to the level of immunosuppression. The whole blood assay used in this study has advantages over the use of isolated lymphocytes in culture medium, as the ratio between plasma protein-bound drugs and their cellular distribution closely reflects physiological conditions, and drugs can be analyzed in clinically relevant concentrations [11]. Moreover, as temperature affects the free concentration of calcineurin inhibitors [12,13], stimulations carried out at 37°C ensured drug distributions comparable with those observed in vivo. The assay may be suitable for pharmacodynamic monitoring, as in vitro immunosuppressive drugs induced a dose-dependent decrease in the percentage of cytokine-producing cells with a low intra- and inter-assay variability. In addition, as exemplified for calcineurin inhibitors, there was a marked interindividual difference in drug-susceptibility. This is of clinical relevance as despite pharmacokinetic monitoring, patients frequently experience toxicity or lack of efficiency [14], which could be balanced by pharmacodynamic assessment of immunosuppressive drugs on T-cell function.

Although both cyclosporine A and tacrolimus exert their immunosuppressive properties by inhibiting the phosphatase activity of calcineurin, major differences exist in terms of molecular structure, side-effect profile [15,16] and clinical outcome [14]. Differences in the inhibitory action on T-cell function were also found when combined with steroids in vitro, as the combined effect with tacrolimus was more pronounced as compared to that of steroids and cyclosporine A. This may be reconciled with the clinical evidence that steroid-resistant rejections are more frequently observed under cyclosporine A than under tacrolimus therapy. In addition, steroid-resistant rejections on a cyclosporine A-based regimen may successfully be treated by sole conversion to tacrolimus, which may be related to its higher inhibitory activity with steroids [17].

To further elucidate potential differences in the effect of immunosuppressive drug-combinations on circadian rhythms of T-cell numbers and functionality, transplanted patients were analyzed before and after the respective morning and evening doses of cyclosporine A and tacrolimus. Steroids were taken in the morning only. In general, circadian rhythm of T-cell numbers and reactivity differed from that in healthy controls [6]. Immunosuppressive drugs altered the circadian rhythm of T-cell counts and reactivity in a dose dependent manner, as short-term transplanted with higher drug-dosages had more pronounced circadian alterations than long-term transplanted patients. The quantitative decrease of CD4 and CD8 T-cell numbers after the morning dose of drugs is in line with results obtained after intravenous application of methylprednisolone, where the nadir of lymphocyte counts was detected around noon 4–8 hours after infusion [18].

Our study is limited by the fact that we did not perform detailed pharmacokinetic profiling to determine potential individual differences in peak levels of drugs after intake over time. However, pharmacodynamic characteristics of T-cell functionality largely corresponded to the pharmacokinetic course of drugs in that tacrolimus had lower peak levels and a flatter blood concentration curve than cyclosporine A. In line with previous reports [19], peak concentrations of both drugs were higher after drug-intake in the morning than in the evening which may result from variations in drug-adsorption, metabolism and elimination [20]. In this respect, the intake of calcineurin inhibitors under fasting conditions in the morning results in more extensive drug-exposure and shorter time to peak concentrations than under fed condition in the evening [21-23]. Hence, apart from the additional activity of steroids in the morning, the differences in T-cell reactivity in the morning and evening may at least in part be due to slight differences in respective drug-levels at the two time points. Additional differences were observed in the pharmacodynamics of cyclosporine A and tacrolimus which were most obvious when T-cell functionality was studied after drug-intake in the evening that was not accompanied by steroids. Whereas the percentage of reactive T-cells remained rather stable or even increased after intake of tacrolimus, their levels showed a significant decrease after intake of cyclosporine A. A similarly pronounced decrease in T-cell frequencies was observed when cyclosporine A was taken together with steroids in the morning. In line with in vitro results, this indicated that steroids did not show any pronounced synergistic activity with cyclosporine A. In contrast, combined treatment of steroids with tacrolimus did show a stronger inhibitory activity as compared to each drug alone, which is illustrated by the decrease in reactive T-cell frequencies after the morning medication, while no decrease was observed after intake of tacrolimus only.

The effectiveness and toxicity of many drugs vary depending on dosing time [24-27] and constitutes the rationale for pharmacotherapy [28]. As animal studies have already shown marked differences in graft survival according to timing of drug-treatment [29,30], knowledge on how diurnal alterations in T-cell function are modulated by immunosuppressive drugs in humans may be translated into clinical practice to identify drug-regimens where circadian dynamics closely reflect natural variations in non-immunocompromized individuals with highest T-cell reactivity around midnight. Based on the results of this study, this functional profile is most closely achieved in long-term transplant recipients on a tacrolimus-based regimen. Our patients were all on a twice daily regimen of tacrolimus. In this context, it is tempting to speculate whether the profile of patients on a once daily regimen may even more closely resemble that of non-immunocompromized individuals. Knowledge of the circadian rhythm of T-cell numbers and their reactivity may also have practical consequences for the use of immune-based assays to study pathogen-specific immune responses in transplanted patients [31-33]. Whereas the kinetics of the healthy controls are considerably stable during routine clinical hours from the morning through the noon [6], reactive CD4 T-cells of short-term transplanted patients may vary on average from 76.19% to −57.38% of the daily mean between morning and noon. When using these assays in clinical practice, this emphasizes the need for standardized timing of blood collection in transplanted patients, especially during routine hours at daytime. Due to the different kinetics of tacrolimus and cyclosporine A, this is even more important for patients on cyclosporine A.

Limitations of our study include the fact that we did not simultaneously analyze clock genes to elucidate whether their expression pattern undergoes similar changes as cell populations or their functionality. The small sample size may be considered as a further limitation. Nevertheless all subjects in the respective groups showed a considerably homogenous pattern of T-cell functionality. An influence of proliferation inhibitors on circadian rhythm of T-cell function described in this study is unlikely, as drugs such as MMF and azathioprine do not have any effect on the early cytokine induction after 6 hours [34]. Hence, further studies with different readouts are needed to monitor individual suppressive activity of anti-proliferative drugs. As a further limitation, we were unable to study the effects of higher doses of cyclosporine A on diurnal variations of T-cell reactivity in short-term transplant recipients, as most of these patients received a tacrolimus-based drug-regimen in our transplant center. Finally, the study was not designed and the sample size is not powered to assess graft survival in relation to individual susceptibility towards immunosuppressive drugs, which should be addressed in future prospective studies.

## Conclusion

The inhibitory effect of immunosuppressive drugs and their combinations may be analyzed on an individual basis. In addition, the two calcineurin inhibitors differentially affect diurnal variations of T-cell reactivity with less variability of T-cell inhibition in the daily course with tacrolimus. Together, this knowledge may improve estimations on the net immunosuppressive effect of a given drug-combination that daily acts in an individual patient, and may help to optimize drug-dosage and timing of drug-intake to individualize immunosuppression with potentially less side-effects.

## Abbreviations

IFN-γ: Interferon-γ
IL-2: Interleukin 2
IQR: Interquartile range
MMF: Mycophenolate mofetile
NK cell: Natural killer cell
SEB: *Staphylococcus aureus* enterotoxin B
